# Nonlinear relationship between triglyceride-glucose index and the risk of prediabetes and diabetes: a secondary retrospective cohort study

**DOI:** 10.3389/fendo.2024.1416634

**Published:** 2024-09-23

**Authors:** Changchun Cao, Haofei Hu, Peng Xiao, Yibang Zan, Xinru Chang, Yong Han, Xiaohua Zhang, Yulong Wang

**Affiliations:** ^1^ Department of Rehabilitation, Shenzhen Dapeng New District Nan’ao People’s Hospital, Shenzhen Second People’s Hospital, Shenzhen, Guangdong, China; ^2^ Department of Nephrology, The First Affiliated Hospital of Shenzhen University, Shenzhen Second People’s Hospital, Shenzhen, Guangdong, China; ^3^ Department of Emergency, The First Affiliated Hospital of Shenzhen University, Shenzhen Second People’s Hospital, Shenzhen, Guangdong, China; ^4^ Department of Rehabilitation, The First Affiliated Hospital of Shenzhen University, Shenzhen Second People’s Hospital, Shenzhen, Guangdong, China

**Keywords:** triglyceride-glucose index, triglyceride, fasting plasma glucose, prediabetes, diabetes, non-linearity

## Abstract

**Background:**

The triglyceride-glucose (TyG) index, recognized for its cost-efficiency and simplicity, serves as an accessible indicator of insulin resistance. Yet, its correlation with the risk of prediabetes and diabetes (Pre-DM/DM) in the Chinese demographic remains uncertain. Consequently, our study explored the association between the TyG index and the development of Pre-DM/DM within the Chinese population.

**Methods:**

The retrospective cohort study was carried out utilizing data from a health screening initiative. The study included 179541 adults over 20 who underwent medical examinations at the Rich Healthcare Group over a period spanning from 2010 to 2016. The correlation between the TyG index and Pre-DM/DM risk was investigated using Cox regression analysis. Furthermore, Cox proportional hazards regression with cubic spline functions and smooth curve fitting was incorporated to explore their non-linear connection.

**Results:**

The mean age of study participants was 41.18 ± 12.20 years old, and 95255 (53.05%) were male. During a median follow-up of 3.01 years, 21281 (11.85%) participants were diagnosed with Pre-DM/DM. After adjusting the potential confounding factors, the results showed that the TyG index was positively correlated with incident Pre-DM/DM (HR: 1.67, 95%CI: 1.62-1.71, P< 0.001). Additionally, a non-linear association was observed between the TyG index and the onset of Pre-DM/DM, with an inflection point identified at 8.73. Hazard ratios (HR) to the left and right of this inflection point were 1.95 (95%CI: 1.86-2.04) and 1.34 (95%CI: 1.27-1.42), respectively. Furthermore, sensitivity analyses confirmed the stability of these findings.

**Conclusion:**

The TyG index exhibited a non-linear positive relationship with the risk of Pre-DM/DM. These findings imply that maintaining the TyG index at a lower, specified threshold may be beneficial in mitigating the onset of Pre-DM/DM.

## Introduction

Diabetes mellitus (DM) has emerged as a prevalent chronic condition on a global scale, witnessing a notable increase in its incidence among chronic diseases worldwide in recent years (1). As the predominant chronic illness, DM exerts a significant economic burden on individuals and healthcare infrastructures. The International Diabetes Federation reported approximately 425 million individuals aged 20 to 79 years were living with DM globally in 2019 (2). Projections suggest an escalation to 642 million by 2045, with Asia accounting for 140.2 million of these cases (2). In addition, the number of individuals with prediabetes, a state defined by glucose levels that are higher than normal but not yet high enough to warrant a diabetes diagnosis, is set to rise significantly (3). It is projected that by 2030, over 470 million people will be living with prediabetes (4). A notable concern is the lack of awareness among individuals with prediabetes regarding their altered glucose metabolism, leading to missed opportunities for preventive interventions. Consequently, an estimated 5% to 10% of individuals with prediabetes progress to DM annually (5). A concerning trend is the decreasing age of onset for both prediabetes and diabetes, with younger populations increasingly affected (1, 6). Individuals of younger age presenting with prediabetes and diabetes frequently experience adverse prognostic outcomes and demonstrate a heightened predisposition towards both cardiovascular and microvascular disorders (7). This change underscores the imperative need for early screening and intervention for prediabetes and diabetes (Pre-DM/DM) to halt the progression of these conditions and mitigate adverse health outcomes.

The triglyceride-glucose (TyG) index, recognized for its cost-efficiency and simplicity, serves as an accessible indicator of insulin resistance (IR) (8), a critical determinant in the pathophysiology of both diabetes and prediabetes. Numerous investigations have highlighted the TyG index’s correlation with an increased cardiovascular disease risk within the general populace (9). Furthermore, it has been posited as a predictive measure for conditions such as arteriosclerosis and coronary artery calcification (10, 11). Currently, several studies have reported the connection between the TyG index and the development of DM. For example, studies in the United States, Korea, and Japan have indicated that the TyG index is an independent risk factor for the development of DM (12–14). Yet, these studies ignored subjects with Pre-DM. The progression from insulin resistance to outright diabetes is not immediate but rather evolves into diminished glucose tolerance, with a significant portion of affected individuals eventually contracting diabetes (15). To bridge this gap in research, our retrospective cohort study endeavored to delineate the precise relationship between the TyG index and the emergence of Pre-DM/DM within a substantial cohort of the Chinese populace.

## Methods

### Data source

The data employed in our research was obtained from the DATADRYAD platform, an online archive offering free access and download capabilities for extensive raw data collection to the scientific community. This platform provided us access to a dataset initially contributed by Chen et al. (16), encompassing information on 211,833 individuals from China. Following Dryad’s guidelines and terms of service, we conducted a secondary analysis of this dataset, which is openly accessible to the public.

### Study population

Authorization for the primary study was granted by the Review Board of the Rich Healthcare Group (16), obviating the need for ethical clearance for our subsequent analysis. The original study and our subsequent research aligned with the Declaration of Helsinki’s principles, adhering to all relevant guidelines and regulatory standards.

**Figure 1:**
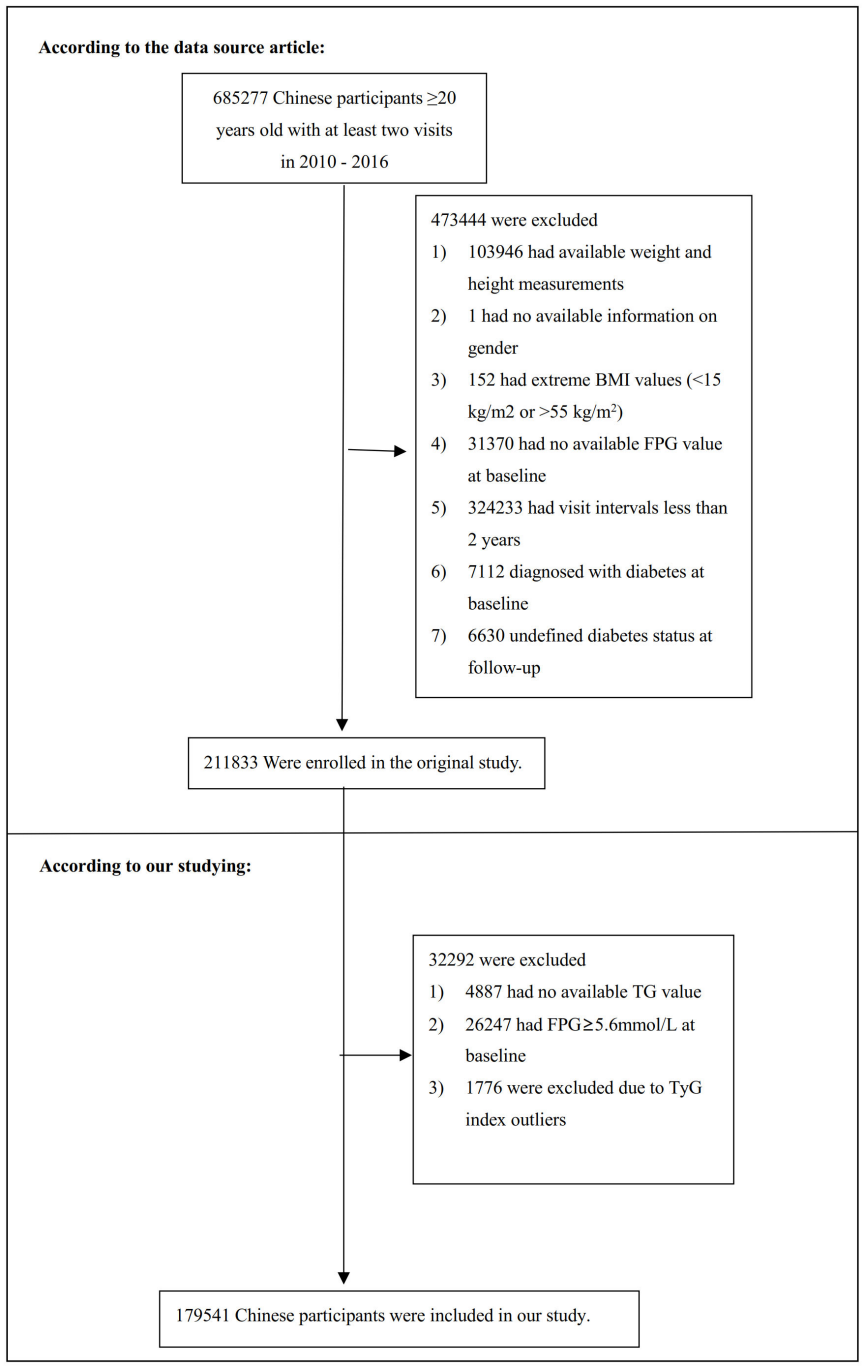
Study population.

The initial research enlisted a cohort comprising 685,277 Chinese adults over 20 years, who had participated in at least two consultations, spanning 11 cities and 32 sites within China. The exclusion criteria were meticulously delineated as follows: (1) a pre-existing diagnosis of diabetes mellitus at the inception of the study; (2) an undefined status of diabetes mellitus during the follow-up phase; (3) an aberrant Body Mass Index (BMI), delineated as a BMI exceeding 55 or falling below 15 kg/m^2; (4) the absence of baseline data pertaining to weight, height, gender, triglycerides (TG), or fasting plasma glucose (FPG); (5) a baseline FPG level surpassing 5.6mmol/L; (6) a follow-up duration less than two years; (7) an abnormal TyG index, identified as three standard deviations above or below the mean. Following the application of these exclusion criteria, a total of 179,541 participants were deemed eligible for inclusion in the study. The architecture and procedural flow of the study were systematically illustrated in **Figure 1**.

### Data collection

Data collection and organization were carried out by personnel who received specific training for these tasks. The original study employed a uniform environment for acquiring lab data, and standardized procedures were adopted for data handling. These trained individuals collected demographic details, covering various metrics such as age, systolic blood pressure (SBP) and diastolic blood pressure (DBP), as well as height and weight. Measurements of height and weight were taken by trained individuals, ensuring participants were in light attire without footwear, and BMI was determined using the formula weight in kilograms divided by the square of height in meters (kg/m^2^). The trained team obtained Blood pressure readings using a traditional mercury sphygmomanometer. Furthermore, this proficient group utilized a Beckman 5800 autoanalyzer for the assessment of clinical parameters, which included FPG, high-density lipoprotein cholesterol (HDL-C), blood urea nitrogen (BUN), TG, serum creatinine (Scr), total cholesterol (TC), alanine aminotransferase (ALT), low-density lipoprotein cholesterol (LDL-C), and aspartate aminotransferase (AST). The TyG index was specifically computed using the Ln[FPG (mg/dL))×(TG (mg/dL)/2) formula. The focal independent variable delineated for this study was the baseline TyG index, whereas the dependent variables were defined as incident Pre-DM/DM mellitus during the follow-up period.

### Outcome measures

The criteria for diagnosing Pre-DM/DM were delineated based on either a fasting blood glucose concentration of 5.6 mmol/L or higher during the follow-up period (17) or through the acquisition of a self-reported diagnosis of diabetes at the time of follow-up assessment.

### Statistical analysis

Statistical analyses were executed employing R software, along with Empower Stats. The TyG index was stratified into quartiles for analytical purposes. The mean and standard deviation represented variables adhering to a normal distribution, whereas the median and interquartile range described variables with a skewed distribution. Categorical variables were represented through the utilization of percentages. The comparison of continuous variables was facilitated through either one-way ANOVA or the Kruskal-Wallis test, and the chi-square test was employed to compare categorical variables. Incidence was articulated in terms of person-years and cumulative incidence rates. The Kaplan-Meier method was applied to compare survival and cumulative event rates.

In our study, the analysis of missing data revealed that there were individuals with missing data for several variables, as follows: 16 individuals (0.01%) for SBP, 1 individual (<0.01%) for TC, 17 individuals (0.01%) for DBP, 79,115 individuals (44.07%) for LDL-C, 105284 (58.64%) individuals for AST,16,142 individuals (8.99%) for BUN, 79622 (44.35%) individuals for HDL-C, 8,278 individuals (4.61%) for Scr, and 1,413 individuals (0.79%) for ALT, 131307 individuals (73.13%) for smoking status, 131307 individuals (73.13%) for drinking status, respectively. To address this, an interpolation model for multiple variables, inclusive of family history of diabetes, ALT, BUN, SBP, smoking status, TC, age, DBP, drinking status, HDL-C, age, LDL-C, AST, Scr, gender, and BMI, was employed utilizing linear regression with ten iterations. The analysis of missing data was predicated on the assumption of randomness in missingness (18, 19).

The influence of each variable on the risk of Pre-DM/DM was evaluated using the univariate Cox regression method. At the same time, the specific association between them was further elucidated through multivariate Cox regression analysis. Additional analyses included a non-adjusted model (Model 1), a minimally-adjusted model (Model 2), and a fully-adjusted model (Model 3) to elucidate the relationship between the TyG index and the risk for Pre-DM/DM. These models were adjusted if the hazard ratios (HR) were altered by at least 10% upon including covariates. We meticulously documented HR and 95% confidence intervals (CI) throughout the study. Due to collinearity with other evaluated factors, TC was excluded from the final multivariate Cox proportional hazards regression equation, as elaborated in **Supplementary Table S1**.

A series of sensitivity analyses were conducted to affirm the robustness of the conclusions. The TyG index was transformed into categorical data based on quartiles for these analyses, and the P for trend was calculated to validate the continuous variable findings of the TyG index and to assess for nonlinearity. The associations of smoking and drinking with an increased incidence of Pre-DM/DM were also scrutinized. For further sensitivity analyses, individuals who were never-drinkers or never-smokers were included to explore the relationship between the TyG index and the risk of Pre-DM/DM. The validity of the results was tested using a generalized additive model (GAM), which incorporated continuous variables as curves within the equation. Moreover, we calculated E-values to rigorously assess the potential presence of unmeasured confounding variables that could affect the observed association between the TyG index and the risk of developing Pre-DM/DM (20).

To explore the nonlinear connection between the TyG index and Pre-DM/DM risk, Cox proportional hazards regression with cubic spline functions and smooth curve fitting was utilized. Upon detecting non-linearity, the inflection point was identified through recursive algorithms, and a two-piecewise Cox proportional hazards regression model was subsequently employed to determine the threshold effect of the TyG index on the incident rates of Pre-DM/DM, informed by the smoothed curve analysis.

Subgroup analyses, considering factors such as family history of diabetes, sex, smoking status, age, BMI, and drinking status, were conducted using the Cox proportional hazard model. Age and BMI were categorized based on clinical cut points (< 60, ≥ 60 years for age; < 24, ≥ 24 kg/m^2^ for BMI). Each stratification underwent a fully adjusted analysis beyond the stratification variables. Interactions between subgroups were verified using a likelihood ratio test. Values of P less than or equal to 0.05 were statistically significant.

## Results

### Baseline characteristics of participants

In this study, we included 179,541 participants who were initially free from Pre-DM/DM. The cohort’s mean age was 41.18 ± 12.20 years, and males constituted 53.05% of the sample. After an average follow-up duration of 3.15 years, 21,281 participants developed Pre-DM/DM. **Table 1** outlines key demographic characteristics, results of laboratory tests, and other relevant variables. Participants were categorized into four quartiles based on their TyG index values. The analysis revealed that individuals in the highest quartile (Q4) exhibited elevated levels of FPG, AST, LDL-C, blood pressure, TG, BMI, ALT, Scr, TC, age, and BUN. This group (Q4 group) also had a higher proportion of males, along with increased rates of family history of diabetes, smoking, and alcohol consumption. Conversely, the lowest quartile group (Q1) showed higher levels of HDL-C in comparison to the remaining groups.

**Table 1:** The baseline characteristics of participants.

HbA1c	Q1(≤7.90)	Q2(7.90 to ≤8.27)	Q3(8.27 to ≤8.68)	Q4(>8.68)	P-value
Participants	44885	44884	44880	44892	
Gender					<0.001
Male	14223 (31.69%)	20754 (46.24%)	26867 (59.86%)	33411 (74.43%)	
Female	30662 (68.31%)	24130 (53.76%)	18013 (40.14%)	11481 (25.57%)	
Age(years)	37.07 ± 9.69	39.85 ± 11.61	42.65 ± 12.86	45.17 ± 12.84	<0.001
Smoking status					<0.001
Current-smoker	3484 (7.76%)	5782 (12.88%)	8178 (18.22%)	11621 (25.89%)	
Ex-smoker	939 (2.09%)	1343 (2.99%)	1829 (4.08%)	2141 (4.77%)	
Never-smoker	40462 (90.15%)	37759 (84.13%)	34873 (77.70%)	31130 (69.34%)	
Drinking status					<0.001
Current-drinker	351 (0.78%)	581 (1.29%)	829 (1.85%)	1171 (2.61%)	
Ex- drinker	3868 (8.62%)	4921 (10.96%)	6130 (13.66%)	7474 (16.65%)	
Never- drinker	40666 (90.60%)	39382 (87.74%)	37921 (84.49%)	36247 (80.74%)	
Family history of diabetes					0.010
No	44078 (98.20%)	43969 (97.96%)	43968 (97.97%)	43957 (97.92%)	
Yes	807 (1.80%)	915 (2.04%)	912 (2.03%)	935 (2.08%)	
SBP (mmHg)	112.02 ± 13.84	115.70 ± 14.98	119.60 ± 15.76	124.22 ± 16.16	<0.001
DBP (mmHg)	69.82 ± 9.48	72.02 ± 9.96	74.49 ± 10.42	77.84 ± 10.84	<0.001
BMI (kg/m^2^)	21.21 ± 2.57	22.24 ± 2.91	23.47 ± 3.11	25.12 ± 3.09	<0.001
ALT (U/L)	13.7 (10.8-18.5)	15.6 (11.8-22.3)	19 (13.6-27.6)	25.3 (17.6-38.2)	<0.001
AST (U/L)	20 (16.35-24.8)	21 (17-26)	22.3 (18-27.93)	25 (20-31.73)	<0.001
HDL-C (mmol/L)	1.45 ± 0.31	1.41 ± 0.30	1.36 ± 0.29	1.27 ± 0.29	<0.001
TG (mmol/L)	0.57 ± 0.13	0.87 ± 0.13	1.25 ± 0.19	2.30 ± 0.81	<0.001
LDL-C (mmol/L)	2.41 ± 0.57	2.60 ± 0.61	2.79 ± 0.66	2.97 ± 0.70	<0.001
TC (mmol/L)	4.27 ± 0.74	4.52 ± 0.79	4.77 ± 0.85	5.10 ± 0.91	<0.001
BUN (mmol/L)	4.52 ± 1.17	4.55 ± 1.18	4.64 ± 1.17	4.74 ± 1.14	<0.001
Scr (umol/L)	64.38 ± 13.94	67.97 ± 16.23	71.21 ± 15.79	74.80 ± 15.05	<0.001
FPG (mmol/L)	4.58 ± 0.50	4.74 ± 0.46	4.84 ± 0.44	4.94 ± 0.42	<0.001

Values are n (%) or mean ± standard deviation or medians (quartile interval).

TyG index, triglyceride-glucose index; SBP, systolic blood pressure; DBP, diastolic blood pressure; BMI, body mass index; ALT, alanine aminotransferase; AST, aspartate aminotransferase; HDL-C, high-density lipoprotein cholesterol; LDL-C, low-density lipoprotein cholesterol; TC, total cholesterol; TG, triglycerides; Scr, serum creatinine; BUN, blood urea nitrogen; FPG, fasting plasma glucose.

### The incidence rate of pre-DM/DM

**Table 2** presents the Pre-DM/DM incidence rates among 179,541 participants throughout the follow-up period. The overall population exhibited a Pre-DM/DM incidence rate of 11.85% (1.70%-12.00%). The incidence rates for the four TyG index quartiles were as follows: 6.16% (5.94%-6.39%), 8.91% (8.65%-9.18%), 12.71% (12.40%-13.02%), and 19.62% (19.25%-19.99%), respectively. Furthermore, the overall population and the four TyG index quartiles reported cumulative incidence rates of 3762.86, 1896.81, 2829.87, 4100.54, and 6329.17 per 100,000 person-years, in that order. Individuals in higher TyG index quartiles demonstrated increased rates of both incidence and cumulative incidence of Pre-DM/DM compared to those in lower quartiles (P for trend < 0.001).

**Table 2:** Incidence rate of prediabetes and diabetes.

TyG index	Participants (n)	Prediabetes and diabetes events (n)	Cumulative incidence (95%CI) (%)	Per 100,000 person-year
Total	179541	21281	11.85 (11.70-12.00)	3762.86
Q1	44885	2767	6.16 (5.94-6.39)	1896.81
Q2	44884	4001	8.91 (8.65-9.18)	2829.87
Q3	44880	5705	12.71 (12.40-13.02)	4100.54
Q4	44892	8808	19.62 (19.25-19.99)	6329.17
P for trend			<0.001	<0.001

TyG index, triglyceride-glucose index; CI, confidence.

**Figure 2:**
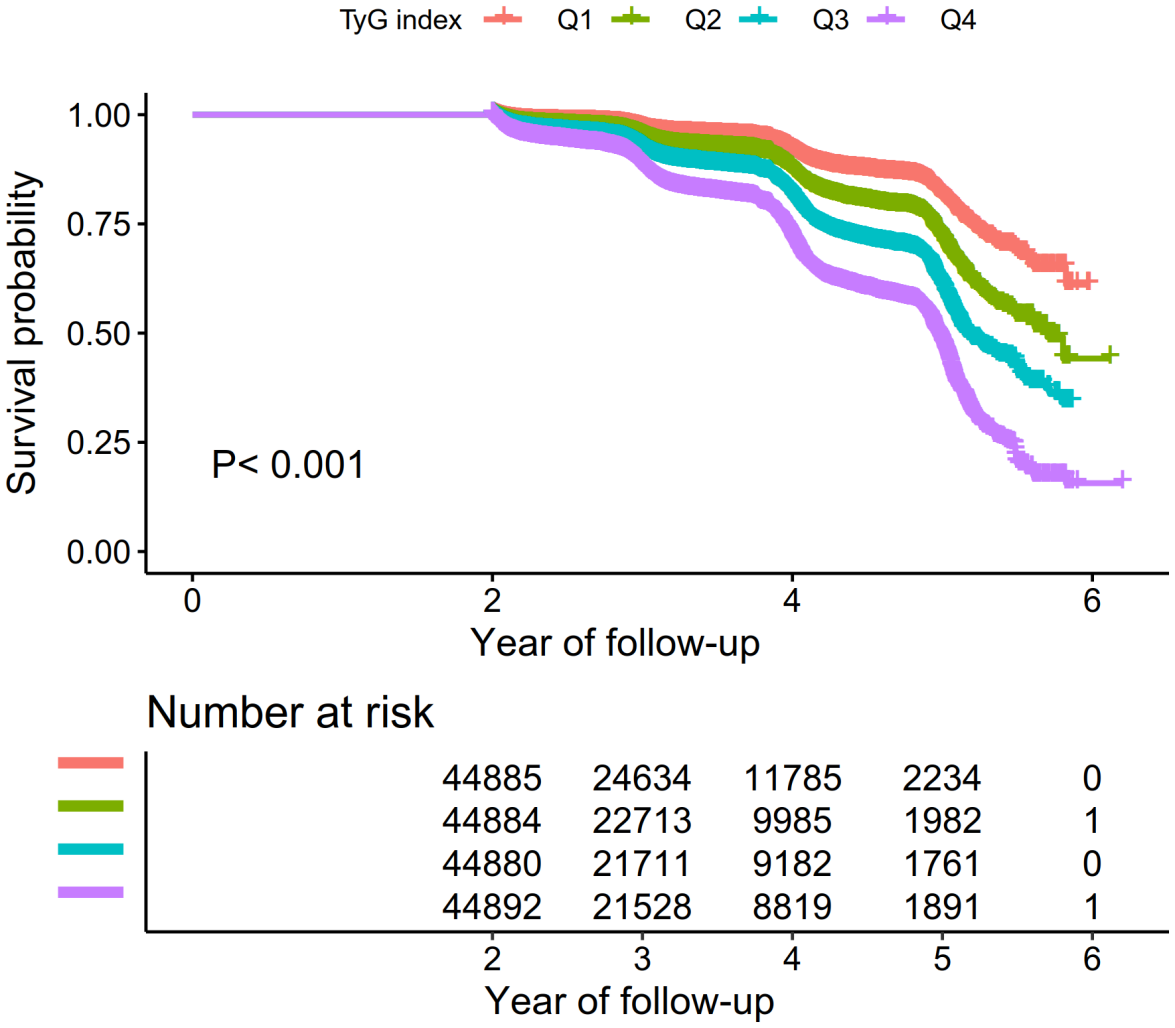
Kaplan–Meier event-free survival curve. Kaplan–Meier analysis of incident prediabetes based on TyG index quartiles (log-rank, P < 0.0001).

**Figure 2** illustrates Kaplan-Meier curves, showcasing the probability of surviving without Pre-DM/DM. There was a significant variance in the risk of developing these conditions across the four TyG index quartiles (P < 0.001), with an ascending TyG index correlating with a reduced probability of avoiding Pre-DM/DM. This signifies that participants within the highest TyG index quartile faced the highest risk of developing Pre-DM/DM.

### Univariate analysis

The findings from the univariate analysis are delineated in **Table 3**. This analysis revealed a statistically significant positive correlation between the risk of developing Pre-DM/DM and several variables, including age, DBP, AST, BUN, BMI, ALT, SBP, TG, FPG, LDL-C, Scr, TC, and TyG index. Conversely, a statistically significant inverse connection was observed between HDL-C and Pre-DM/DM risk. Additionally, never drinkers, smokers, and females demonstrated a significantly lower risk of Pre-DM/DM development.

**Table 3:** The results of the univariate analysis.

	Statistics	HR (95%CI)	P value
Gender			<0.001
Male	95255 (53.05%)	ref	
Female	84286 (46.95%)	0.64 (0.62, 0.66)	
Age(years)	41.18 ± 12.20	1.03 (1.03, 1.04)	<0.001
Smoking status			
Current-smoker	29065 (16.19%)	ref	
Ex-smoker	6252 (3.48%)	0.86 (0.80, 0.93)	<0.001
Never-smoker	144224 (80.33%)	0.70 (0.68, 0.72)	<0.001
Drinking status			
Current-drinker	2932 (1.63%)	ref	
Ex- drinker	22393 (12.47%)	0.74 (0.67, 0.81)	<0.001
Never- drinker	154216 (85.89%)	0.65 (0.59, 0.71)	<0.001
Family history of diabetes			0.1026
No	175972 (98.01%)	ref	
Yes	3569 (1.99%)	1.07 (0.99, 1.17)	
SBP (mmHg)	117.88 ± 15.87	1.03 (1.02, 1.03)	<0.001
DBP (mmHg)	73.54 ± 10.61	1.03 (1.03, 1.03)	<0.001
BMI (kg/m2)	23.01 ± 3.27	1.13 (1.12, 1.13)	<0.001
ALT (U/L)	23.24 ± 21.75	1.00 (1.00, 1.00)	<0.001
AST (U/L)	23.64 ± 12.14	1.01 (1.00, 1.01)	<0.001
HDL-C (mmol/L)	1.37 ± 0.31	0.78 (0.75, 0.82)	<0.001
TG (mmol/L)	1.25 ± 0.78	1.40 (1.39, 1.42)	<0.001
LDL-C (mmol/L)	2.69 ± 0.67	1.28 (1.26, 1.31)	<0.001
TC (mmol/L)	4.67 ± 0.88	1.22 (1.20, 1.24)	<0.001
BUN (mmol/L)	4.61 ± 1.17	1.14 (1.13, 1.15)	<0.001
Scr (umol/L)	69.59 ± 15.76	1.01 (1.01, 1.01)	<0.001
FPG (mmol/L)	4.77 ± 0.48	5.64 (5.45, 5.84)	<0.001
TyG index	8.30 ± 0.57	2.26 (2.21, 2.32)	<0.001

TyG index, triglyceride-glucose index; SBP, systolic blood pressure; DBP, diastolic blood pressure; BMI, body mass index; ALT, alanine aminotransferase; AST, aspartate aminotransferase; HDL-C, high-density lipoprotein cholesterol; LDL-C, low-density.

lipoprotein cholesterol; TC, total cholesterol; TG, triglycerides; Scr, serum creatinine; BUN, blood urea nitrogen; FPG, fasting plasma glucose; HR, hazard ratios; CI, confidence interval; Ref, reference.

### The relationship between the TyG index and pre-DM/DM

Utilizing Cox proportional hazard regression analyses, **Table 4** delineates the relationship between the TyG index and the incidence of Pre-DM/DM. The HR with a 95%CI associating the TyG index with the risk of developing Pre-DM/DM was calculated as 2.26 (2.21-2.32) in the model without any adjustments (Model 1). When the model was minimally adjusted to account for BMI, family history of diabetes, DBP, sex, smoking status, SBP, age, and drinking status, the HR (95% CI) shifted to 1.61 (1.57, 1.65). In the fully adjusted model (Model 3), which was further adjusted for Scr, HDL-C, ALT, BUN, LDL-C, and AST, the HR (95% CI) was 1.67 (1.62, 1.71). This indicated a 67% escalation in the risk of developing Pre-DM/DM with each incremental increase in the TyG index.

**Table 4:** Relationship between the TyG index and incident prediabetes and diabetes in different models.

Variable	Model 1 (HR,95%CI, P)	Model 2 (HR, 95%CI, P)	Model 3 (HR, 95%CI, P)	Model 4 (HR, 95%CI, P)
TyG index	2.26 (2.21, 2.32) <0.001	1.61 (1.57, 1.65) <0.001	1.67 (1.62, 1.71) <0.001	1.67 (1.62, 1.72) <0.001
TyG index (quartile)				
Q1	ref	ref	ref	ref
Q2	1.60 (1.52, 1.68) <0.001	1.30 (1.24, 1.37) <0.001	1.33 (1.27, 1.40) <0.001	1.31 (1.25, 1.38) <0.001
Q3	2.41 (2.30, 2.52) <0.001	1.63 (1.56, 1.71) <0.001	1.70 (1.62, 1.78) <0.001	1.67 (1.59, 1.75) <0.001
Q4	3.74 (3.59, 3.91) <0.001	2.09 (1.99, 2.20) <0.001	2.22 (2.11, 2.33) <0.001	2.19 (2.08, 2.31) <0.001
P for trend	<0.001	<0.001	<0.001	<0.001

Model 1: we did not adjust for other covariates.

Model 2: we adjusted for gender, age, SBP, DBP, family history of diabetes, drinking status, smoking status, and BMI.

Model 3: we adjusted for gender, age, SBP, DBP, family history of diabetes, drinking status, smoking status, BMI, HDL-C, LDL-C, AST, ALT, Scr, and BUN.

Model 4: All covariates listed in **Table 1** were adjusted. However, continuous covariates were adjusted as nonlinearity.

HR, hazard ratios; CI, confidence interval; Ref, reference; TyG index, triglyceride-glucose index.

### The results of sensitivity analysis

To ascertain the reliability of our findings, we conducted a sensitivity analysis. This involved reclassifying the TyG index from a continuous to a categorical variable, which was subsequently reintegrated into the analysis as such. The transition of the TyG index into a categorical framework revealed a non-uniform trend, hinting at a potential nonlinear relationship between the TyG index and the risk of Pre-DM/DM. As depicted in **Table 4**, the outcomes derived from the GAM aligned with those obtained from the comprehensively adjusted model (Model 4, HR=1.67, 95%CI: 1.62-1.72). Furthermore, we calculated an E-value to evaluate the robustness of our findings against the influence of unobserved confounding variables. The derived E-value of 2.73, which surpasses the relative risk of 2.00 linked with unmeasured confounders and the TyG index, underscores the minimal impact that unaccounted or unknown confounding factors might have on the established association between the TyG index and the incidence of Pre-DM/DM.

In addition, our study conducted a sensitivity analysis focusing on individuals who have never consumed alcohol. This analysis uncovered a significant link between the TyG index and an increased likelihood of developing Pre-DM/DM, even after adjusting for other influencing factors (HR=1.70, 95%CI: 1.65-1.75), as shown in **Table 5**. Furthermore, we extended our sensitivity analysis to include individuals who have never smoked. These results consistently demonstrated a positive correlation between the TyG index and the risk of Pre-DM/DM after accounting for potential confounders (HR=1.70, 95%CI: 1.65-1.76), also detailed in **Table 5**. The outcomes of these sensitivity analyses support the robustness of our findings.

**Table 5:** Relationship between the TyG index and prediabetes and diabetes in different sensitivity analyses.

Exposure	Model 5 (HR,95%CI, P)	Model 6 (HR,95%CI, P)
TyG index	1.70 (1.65, 1.75) <0.001	1.70 (1.65, 1.76) <0.001
TyG index (quartile)		
Q1	ref	ref
Q2	1.36 (1.29, 1.44) <0.001	1.39 (1.31, 1.46) <0.001
Q3	1.73 (1.64, 1.82) <0.001	1.75 (1.66, 1.85) <0.001
Q4	2.29 (2.17, 2.42) <0.001	2.28 (2.15, 2.41) <0.001
P for trend	<0.001	<0.001

Model 5 was sensitivity analysis in participants with never-drinker. We adjusted gender, age, SBP, DBP, family history of diabetes, smoking status, BMI, HDL-C, LDL-C, AST, ALT, Scr, and BUN.

Model 6 was sensitivity analysis in participants with never-smoker. We adjusted gender, age, SBP, DBP, family history of diabetes, drinking status, BMI, HDL-C, LDL-C, AST, ALT, Scr, and BUN.

HR, hazard ratios; CI, confidence interval; Ref, reference; TyG index, triglyceride-glucose index.

### The nonlinear relationship between the TyG index and pre-DM/DM

**Figure 3:**
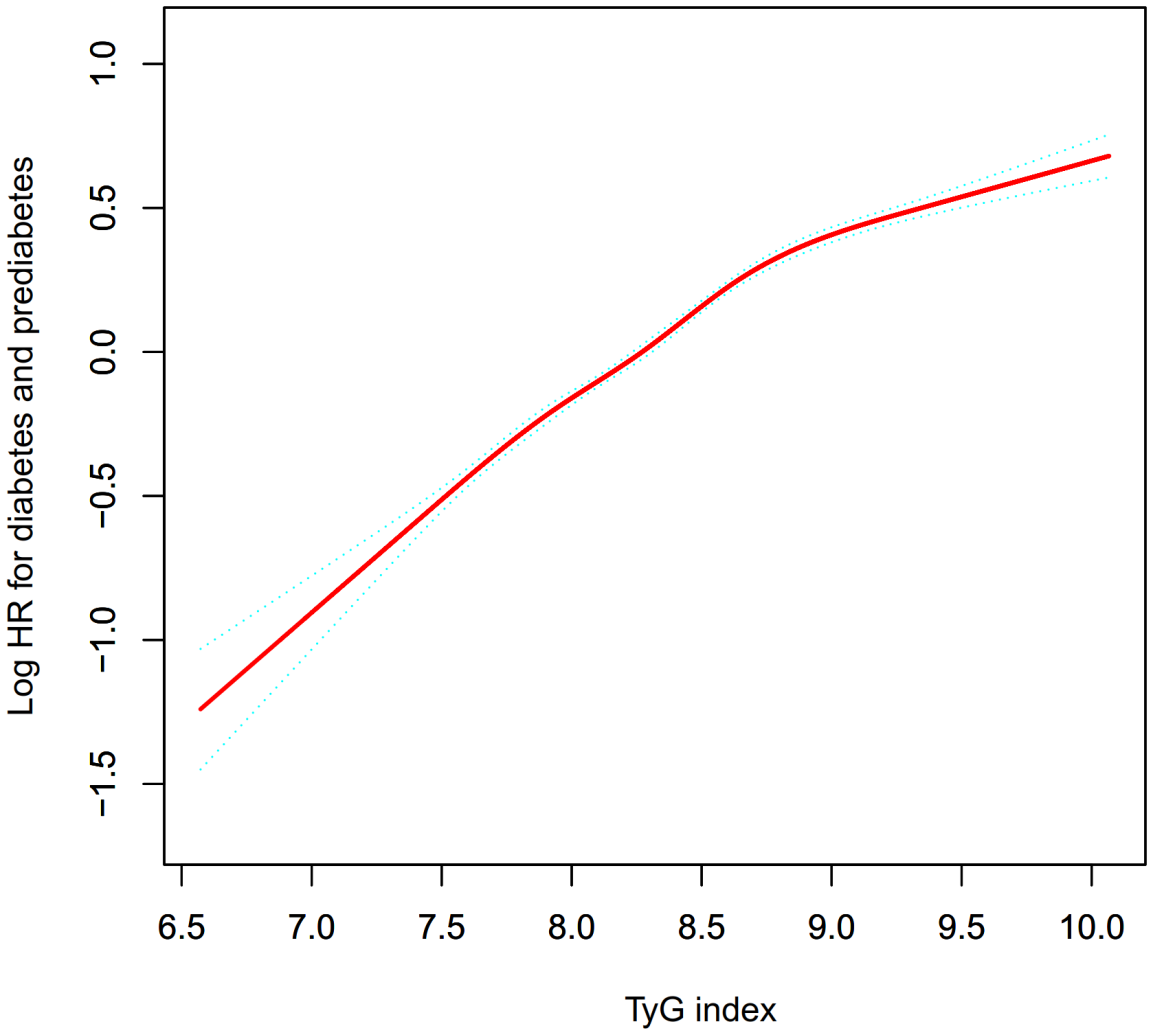
The nonlinear relationship between the TyG index and incident prediabetes. A nonlinear relationship between them was detected after adjusting for gender, age, SBP, DBP, family history of diabetes, drinking status, smoking status, BMI, HDL-C, LDL-C, AST, ALT, Scr, and BUN.

**Figure 3** demonstrates a non-linear connection between the TyG index and the risk of onset for Pre-DM/DM. Upon controlling for potential confounding variables, the relationship between the TyG index and the likelihood of progression to Pre-DM/DM was non-linear (**Table 6**). Initially, the inflection point for the TyG index was identified as 8.73 using a recursive method. Subsequently, a segmented Cox proportional hazards regression approach was employed to evaluate the HR and CI on either side of this pivotal value. To the right of this inflection point, the HR stood at 1.95 (95% Confidence Interval: 1.86-2.04), and to the left, it decreased to 1.34 (95% Confidence Interval: 1.27-1.42).

**Table 6:** The result of the two-piecewise Cox proportional hazards regression model.

Incident prediabetes and diabetes	HR (95%CI)	P
Fitting model by standard Cox proportional hazards regression	1.67 (1.62, 1.71)	<0.001
Fitting model by two-piecewise Cox proportional hazards regression		
Inflection points of the TyG index	8.73	
≤8.73	1.95 (1.86, 2.04)	<0.001
>8.73	1.34 (1.27, 1.42)	<0.001
P for log-likelihood ratio test	<0.001	

We adjusted for gender, age, SBP, DBP, family history of diabetes, drinking status, smoking status, BMI, HDL-C, LDL-C, AST, ALT, Scr, and BUN.

HR, hazard ratios; CI, confidence interval; Ref, reference; TyG index, triglyceride-glucose index.

### Subgroup analysis

We assessed the interplay among diverse variables and their impact on the relationship between the TyG index and the risk of Pre-DM/DM across various exploratory subgroups (**Table 7**). The findings indicated that a familial history of diabetes did not alter the association between the TyG index and the risk of developing Pre-DM/DM. Furthermore, our analysis revealed a more pronounced association within subgroups, namely never drinkers, females, never smokers, people with BMI< 24 kg/m^2^, and age <60 years.

**Table 7:** Effect size of the TyG index on prediabetes and diabetes in prespecified and exploratory subgroups.

Characteristic	No of patients	HR (95%CI)	P value	P for interaction
Age(years)				<0.001
<60	161481	1.83 (1.77, 1.88)	<0.001	
≥60	18060	1.39 (1.30, 1.47)	<0.001	
Gender				<0.001
Male	95255	1.54 (1.49, 1.59)	<0.001	
Female	84286	1.95 (1.86, 2.03)	<0.001	
Smoking status				0.001
Current-smoker	29065	1.56 (1.48, 1.64)	<0.001	
Ex-smoker	6252	1.46 (1.29, 1.64)	<0.001	
Never-smoker	144224	1.71 (1.66, 1.77)	<0.001	
Drinking status				0.005
Current drinker	2932	1.50 (1.28, 1.76)	<0.001	
Ever drinker	22393	1.52 (1.42, 1.63)	<0.001	
Never drinker	154216	1.70 (1.65, 1.75)	<0.001	
Family history of diabetes				0.881
No	175972	1.66 (1.62, 1.71)	<0.001	
Yes	3569	1.68 (1.46, 1.94)	<0.001	
BMI (kg/m^2^)				<0.001
<24	114461	1.92 (1.85, 2.00)	<0.001	
≥24	65080	1.57 (1.52, 1.63)	<0.001	

Note 1: The above model was adjusted for gender, age, SBP, DBP, family history of diabetes, drinking status, smoking status, BMI, HDL-C, LDL-C, AST, ALT, Scr, and BUN.

Note 2: The model was not adjusted for the stratification variable in each case.

## Discussion

Our retrospective analysis indicated a positive correlation between elevated TyG index levels and an increased likelihood of developing Pre-DM/DM. Moreover, upon identifying an inflection point, we noted different correlations on either side of this point regarding the TyG index and Pre-DM/DM risk. Notably, the link between the TyG index and the risk of Pre-DM/DM was more pronounced among never-drinkers, females, never-smokers, individuals with BMI< 24 kg/m^2^, and ages <60 years.

The TyG index, which combines FPG and TG, has been recognized as a potential substitute for measuring insulin resistance (8). Compared to traditional insulin resistance markers, such as the homeostasis model assessment of insulin resistance (HOMA-IR), the TyG index is a practical, cost-effective, and reliable tool for assessing insulin resistance, particularly advantageous in low-resource settings. Research conducted in the past has investigated the connection between the TyG index and diabetes, consistently revealing a positive correlation between the index and the occurrence of diabetes. A comprehensive review of 15 cohort studies underscored a notable direct link between the TyG index and the incidence of diabetes, positing the TyG index as a viable marker for pinpointing those at heightened risk for diabetes (21). A particular investigation in South Korea established a significant connection between the TyG index and insulin resistance in diabetes, proving it superior to the homeostatic model assessment of insulin resistance for forecasting diabetes in the youth demographic (14). In a separate analysis conducted by Chen et al. (22) among Chinese adults, it was observed that for every standard deviation increment in the TyG index, the likelihood of diabetes surged by 22% (HR=1.22, 95%CI: 1.14-1.31). Nevertheless, individuals with prediabetes, who face a significant risk of evolving into diabetic patients, have traditionally been overlooked. It is only in recent times that the scientific community has initiated investigations into the link between the TyG index and prediabetes. Characterized by a chronic state of moderate hyperglycemia without symptoms, prediabetes has the potential to advance to diabetes if it remains unidentified (23). Consequently, our research treated both Pre-DM/DM as outcome variables in order to scrutinize their association with the TyG index. By doing so, our research augments the body of existing evidence, corroborating the theory that an increased TyG index is intricately linked with the heightened risk of emerging Pre-DM/DM. This insight is valuable for the early identification of Pre-DM/DM and the implementation of prompt interventions.

Moreover, our research identified a non-linear association between the TyG index and the likelihood of developing Pre-DM/DM. Adjusting for potential confounders revealed an inflection point in the TyG index at 8.73. Below this threshold, an increment of one unit in the TyG index corresponded to a 95% heightened risk of Pre-DM/DM (HR=1.95, 95%CI: 1.86-2.04). Conversely, above this inflection point, each additional unit in the TyG index was linked to a 34% increased risk (HR=1.34, 95%CI: 1.27-1.42). Therefore, a reduction in the TyG index is associated with a diminished risk of Pre-DM/DM. It is important to highlight that the decrease in risk becomes more pronounced when the TyG index falls below 8.73. On the other hand, the reduction in risk decelerates when the index surpasses 8.73.

Numerous studies robustly support the TyG index as a reliable marker of hyperinsulinemia and insulin resistance (24, 25). Hyperinsulinemia, characterized by elevated levels of insulin in the bloodstream, is frequently associated with insulin resistance and plays a crucial role in the pathophysiology of diabetes mellitus (26). This insulin-resistant state necessitates higher insulin levels for glucose homeostasis, resulting in the overproduction and persistence of insulin in the circulation (26). Hyperinsulinemia initiates and perpetuates a cycle of metabolic disturbances that significantly contribute to disease progression (26, 27). The compensatory hypersecretion of insulin in response to insulin resistance exacerbates pancreatic β-cell dysfunction over time, ultimately leading to β-cell exhaustion and the onset of hyperglycemia (28). In addition, the implications of hyperinsulinemia extend beyond glycemic control, influencing both cardiovascular and non-cardiovascular health outcomes. Cardiovascularly, hyperinsulinemia is an established risk factor for atherosclerosis, hypertension, and coronary artery disease (29–31). Insulin exerts atherogenic effects by promoting vascular smooth muscle cell proliferation, enhancing lipoprotein retention within arterial walls, and inducing endothelial dysfunction (30). Moreover, insulin resistance and hyperinsulinemia are associated with an adverse lipid profile, characterized by elevated levels of LDL-C and decreased HDL-C, further exacerbating cardiovascular risk (32). Regarding non-cardiovascular adverse events, the elevated insulin levels observed in hyperinsulinemic states have been implicated in depression, and non-alcoholic fatty liver disease (33–35). Hyperinsulinemia exacerbates inflammation and oxidative stress, alters norepinephrine levels in the brain’s sympathetic system, and disrupts neurotransmitter metabolism and synaptic plasticity, thereby contributing to the development of depression by inhibiting serotonin, dopamine, melatonin, and glutamate signaling (33). Additionally, hyperinsulinemia can contribute to hepatic steatosis and fibrosis by promoting lipogenesis and inflammation within the liver (35). Hyperinsulinemia associated with insulin resistance is a fundamental component in the pathophysiology of diabetes mellitus and plays a decisive role in a broad spectrum of related adverse events. It is imperative to implement interventions at the stage of insulin resistance and hyperinsulinemia, which frequently precede the onset of prediabetes and diabetes by several years (27, 36). Screening for insulin resistance and hyperinsulinemia in at-risk populations using the TyG index can significantly improve patient prognosis by enabling highly preventive interventions.

Our study highlights the significant association between the TyG index and the risk of developing Pre-DM and T2DM in a cohort of 179541 adults. The findings underscore the potential of the TyG index as a valuable, non-invasive marker for early identification of individuals at higher risk of progressing to PreDM and DM. Considering its simplicity and cost-effectiveness in comparison to other measures of insulin resistance, such as the homeostasis model assessment of insulin resistance, the TyG index can be readily incorporated into routine clinical practice. This can facilitate timely interventions aimed at lifestyle modification, dietary changes, and possibly pharmacological treatment to mitigate risk and delay or prevent the onset of diabetes. Early identification and management of high-risk individuals using the TyG index can substantially improve patient outcomes and reduce the burden of diabetes-related complications.

In addition, the TyG index, as a surrogate marker for insulin resistance, has been evaluated in numerous meta-analysis studies across a variety of diseases, demonstrating its wide range of applications. A systematic review and meta-analysis of thirty studies with 772,809 participants revealed that higher TyG index levels were associated with an increased risk of heart failure (37). Another meta-analysis encompassing twelve cohort studies with 6,354,990 participants found that higher TyG index levels were significantly associated with an increased incidence of cardiovascular disease (38). In addition, higher TyG index levels were also related to an increased risk of atrial fibrillation, arterial stiffness, coronary artery disease, and ischemic stroke (39–42). The meta-analyses above reinforce our findings and highlight the TyG index’s robustness in predicting not only diabetes and prediabetes but also its broader application in various diseases. This adds to the growing body of evidence supporting the TyG index as an indispensable tool in clinical and public health settings, offering significant advantages in terms of accessibility and predictive power.

The precise biological pathways connecting the TyG index to the susceptibility of developing diabetes and prediabetes are not fully understood, yet they likely involve insulin resistance (15, 43). Evidence has underscored the pivotal function of insulin resistance in the emergence and advancement of both diabetes and prediabetes. Constituted by FPG and TG, the TyG index serves as an indicator. The levels of FPG mirror the liver’s insulin sensitivity and the pancreatic secretion of insulin, with elevated FPG levels correlating with a heightened diabetes risk even in individuals with normoglycemia (44, 45). Furthermore, the efficacy of TG as an insulin resistance marker has received substantial validation in prior research (46–48). Consequently, the connection between the TyG index and an increased likelihood of diabetes and prediabetes development could be attributed to the interplay among FPG, TG, and insulin resistance.

Our investigation possesses several notable strengths. Firstly, it delves into the non-linear relationship between the TyG index and the incidence of Pre-DM/DM. Secondly, to address the issue of incomplete data, we employed multiple imputation techniques, enhancing the reliability of our statistical analyses and reducing potential bias arising from omitted variables. Furthermore, we undertook a comprehensive set of sensitivity analyses to affirm the validity of our findings. These included transforming the TyG index into discrete categories, applying a GAM to incorporate continuous covariates as non-linear variables, and reassessing the link between the TyG index and the risk of Pre-DM/DM while excluding individuals who consume alcohol or tobacco.

Our research comes with certain constraints. Initially, the fact that our cohort consisted solely of Chinese individuals suggests a need for further exploration into the link between the TyG index and the risk of Pre-DM/DM in diverse populations. Additionally, glucose metabolism disorders were operationalized within our study parameters as an FPG level equal to or exceeding 5.6 mmol/L or the self-reporting of diabetes by participants during the follow-up period. This definition did not incorporate the assessment of glycosylated hemoglobin levels or the administration of a 2-hour oral glucose tolerance test. As a result, such methodological delineations may have led to a conservative estimation of Pre-DM/DM incidence, potentially underrepresenting the true prevalence within the studied cohort. Furthermore, as with any observational study, the potential for unaddressed or unobserved confounding variables, such as dietary habits and physical activity levels, to influence the findings cannot be completely eliminated. However, through the application of the E-value, we determined it unlikely that these unmeasured confounders significantly impacted our results. Moving forward, we aim to collaborate with international scholars to gather more detailed information on diet and exercise habits and to examine the validity of our findings across populations with varying genetic predispositions. Finally, our study lacked data related to cardiovascular and non-cardiovascular events. In the future, we will design our own study to collect cardiovascular and non-cardiovascular events and further analyze the relationship between TyG and the risk of cardiovascular and non-cardiovascular events.

## Conclusion

This research establishes a positive, non-linear link between the TyG index and the onset of Pre-DM/DM among prediabetic Chinese adults. Notably, a TyG index at or below 8.73 was strongly correlated with an increased risk of Pre-DM/DM. The results underscore the significance of the TyG index as an effective tool for forecasting the likelihood of Pre-DM/DM, indicating its prospective utility in healthcare settings for evaluating risk and steering preventative measures.

## Data Availability

The datasets presented in this study can be found in online repositories. The names of the repository/repositories and accession number(s) can be found below: https://datadryad.org/stash/dataset/doi:10.5061%2Fdryad.ft8750v.
