# Supplementary material for: Nonlinear relationship between triglyceride-glucose index and the risk of prediabetes and diabetes: a secondary retrospective cohort study
Source: Front Endocrinol (Lausanne). 2024 Sep 23;15:1416634. doi: 10.3389/fendo.2024.1416634 (PMC11460547; doi:10.3389/fendo.2024.1416634)
Supplement: Supplementary file 1 [file Table1.docx]

**Table S1** The results of the collinearity screening

|  | Step 1 | Step 2 |
| --- | --- | --- |
| Gender | 2.6 | 2.6 |
| Age(years) | 1.3 | 1.3 |
| Drinking status | 1.1 | 1.1 |
| Smoking status | 1.3 | 1.3 |
| Family history of diabetes | 1 | 1 |
| SBP (mmHg) | 2.1 | 2.1 |
| DBP (mmHg) | 2 | 2 |
| BMI (kg/m^2^) | 1.5 | 1.4 |
| ALT (U/L) | 3.3 | 3.3 |
| AST (U/L) | 2.9 | 2.9 |
| HDL-C (mmol/L) | 1.4 | 1.2 |
| LDL-C (mmol/L) | 5.3 | 1.1 |
| TC (mmol/L) | 5.9 | NA |
| BUN (mmol/L) | 1.2 | 1.2 |
| Scr (umol/L) | 2.1 | 2.1 |

SBP systolic blood pressure, DBP diastolic blood pressure, BMI body mass index, ALT alanine aminotransferase, AST aspartate

aminotransferase, HDL-C high-density lipoprotein cholesterol, LDL-C low-density lipoprotein cholesterol, TC total cholesterol,

Scr serum creatinine, BUN blood urea nitrogen
